# The Causes and Consequences of miR-503 Dysregulation and Its Impact on Cardiovascular Disease and Cancer

**DOI:** 10.3389/fphar.2021.629611

**Published:** 2021-03-08

**Authors:** Yanjing He, Yin Cai, Pearl Mingchu Pai, Xinling Ren, Zhengyuan Xia

**Affiliations:** ^1^ Department of Anesthesiology, The University of Hong Kong, Hong Kong, China; ^2^ Department of Health Technology and Informatics, The Hong Kong Polytechnic University, Hong Kong, China; ^3^ Department of Medicine, The University of Hong Kong - Shenzhen Hospital, Shenzhen, China; ^4^ Department of Medicine, The University of Hong Kong - Queen Mary Hospital, Hong Kong, China; ^5^ Department of Respiratory Medicine, Shenzhen University General Hospital, Shenzhen, China; ^6^ State Key Laboratory of Pharmaceutical Biotechnology, Department of Medicine, The University of Hong Kong, Hong Kong, China; ^7^ Department of Anesthesiology, Affiliated Hospital of Guangdong Medical University, Zhanjiang, China

**Keywords:** microRNAs, microRNA-503, cardiovascular disease, cancer, angiogenesis, oxidative stress

## Abstract

microRNAs (miRs) are short, non-coding RNAs that regulate gene expression by mRNA degradation or translational repression. Accumulated studies have demonstrated that miRs participate in various biological processes including cell differentiation, proliferation, apoptosis, metabolism and development, and the dysregulation of miRs expression are involved in different human diseases, such as neurological, cardiovascular disease and cancer. microRNA-503 (miR-503), one member of miR-16 family, has been studied widely in cardiovascular disease and cancer. In this review, we summarize and discuss the studies of miR-503 *in vitro* and *in vivo*, and how miR-503 regulates gene expression from different aspects of pathological processes of diseases, including carcinogenesis, angiogenesis, tissue fibrosis and oxidative stress; We will also discuss the mechanisms of dysregulation of miR-503, and whether miR-503 could be applied as a diagnostic marker or therapeutic target in cardiovascular disease or cancer.

## Introduction

microRNAs (miRs) are small non-coding RNAs with 19-23 nucleotides (nt) in length that modulate target genes through mRNA degradation or translational repression (Bartel, 2004; Ahmed et al., 2018). miRs biogenesis includes canonical and non-canonical pathways, and has been summarized in several reviews (Bartel, 2004; O’Brien et al., 2018). miR genes are transcribed by RNA polymerase II to generate primary miR transcripts (pri-miRs), which is cleaved by RNase III endonuclease Drosha to produce about 60–70 nt stem loop intermediate, termed miR precursor, or the pre-miR. The pre-miR is transported by Ran-GTP and export receptor Exportin-5 from nucleus to the cytoplasm. In canonical pathway of miRs genesis, pre-miR is processed by another RNase III endonuclease, Dicer, to produce miR:miR duplex comprising the mature miR strand and its complementarystrand. The mature miR is loaded into the cytoplasmic RNA-induced silencing complex (RISC) where it binds to the 3′-untranslated region (UTR) of target mRNAs with complementary sites, resulting in mRNA degradation or its translational repression.

miRs are implicated in various biological processes, including cell differentiation (Chen et al., 2004), proliferation, apoptosis (Brennecke et al., 2003), metabolism (Joacim et al., 2008), and development (Gerhard et al., 2006), and in pathological situations like hypoxic cellular injury and repairing (Chen et al., 2013; Ge L. et al., 2019). Over 1/3 of human protein–coding genes are modulated by miRs (Lewis et al., 2005). Growing evidence has demonstrated that numerous miRs are dysregulated in human diseases. Some miRs are upregulated, such as miR-25 in heart failure (Christine et al., 2014), miR-133a in acute myocardial infarction (Wang et al., 2013), miR-155-5p in chronic kidney disease and nocturnal hypertension (Klimczak et al., 2017) are enhanced, while some miRs are downregulated in other diseases, such as miR-320a in arrhythmogenic cardiomyopathy (Sommariva et al., 2017), both miR-133 and miR-1 in hypertrophic cardiomyopathy (Alessandra et al., 2007), miR-107 in Alzheimer’s disease (Wang et al., 2008), and miR-503-5p in non-small cell lung cancer (Yang et al., 2014) are reduced. Therefore, therapies that inhibit miR function or restore its function have been developed for human diseases (Couzin, 2008; Manel, 2011).

microRNA-503 (miR-503) is an intragenic miR located on the chromosomal location Xq26.3 and clustered with miR-424 in human (Griffiths-Jones et al., 2008). It belongs to the miR-16 family (Caporali and Emanueli, 2011), and was first identified in human retinoblastoma tissues by microRNA microarray analysis (Zhao et al., 2009). The other members of miR-16 family are miR-15a, miR-15b, miR-16, miR-195, miR-424 and miR-497, miR-16 family have similarity in their individual seed sequences (Caporali and Emanueli, 2011). According to gene nomenclature, in human, there are two different mature miR-503, one is hsa-miR-503-5p, derived from 5′ends of pre-miR-503, the other is hsa-miR-503-3p, derived from 3′ends of pre-miR-503, both are excised from the same precursor microRNA (Griffiths-Jones et al., 2006), and thus, miR-503 rather than miR-503-5p is usually used. Although these “5p” and “3p” miRs are originated from a single primary transcript, they have different sequences and mRNA targets, therefore, they have different, even converse roles in biological function (Almeida et al., 2012). Accumulated evidence has also shown that miR-503 modulates various biological processes, and the dysregulation of miR-503 is associated with human diseases. In this review, we will mainly focus on describing the roles of miR-503 in human diseases, especially cardiovascular disease and cancer, from different aspects of physiological or pathological processes, including cell differentiation, proliferation, apoptosis, carcinogenesis, angiogenesis, tissue fibrosis and oxidative stress.

### Physiological Roles of miR-503 in Regulating Cell Differentiation

The relationship between cell proliferation and cell differentiation is complicated, and the imbalance of cell proliferation and differentiation can result in a variety of diseases such as cancer (Ruijtenberg and van Den Heuvel, 2016). MiR-424/-503 was found to a polycistronic microRNA cluster, the loci of them were separated by 383 bases on the genome (Forrest et al., 2009). In the differentiation of monoblasts into monocytes, overexpression of either miR-424 or miR-503 can induce cell cycle arrest at G1 phase by targeting an anti-differentiative miR-9 and several regulators of cell cycle, therefore, both miR-424 and miR-503 can partially promote monocytic differentiation and inhibit cell proliferation at G1 phase in the cell cycle (Forrest et al., 2009). Consistent with this, induction of G1 phase cell cycle arrest, which is mainly caused by the suppression of cyclin-dependent-kinase 2 (cdk2), is a critical step during myogenesis (Sarkar et al., 2010). miR-424 and miR-503 were induced in the differentiation of myoblasts into myotubes and promotedcdk2 inhibition by downregulating Cdc25A (Sarkar et al., 2010). Cdc25A, a phosphatase that is the target gene of both miR-424 and miR-503, can get rid of the inhibitory phosphorylation on cdk2, and thus promote cell cycle progress (Sarkar et al., 2010). In another study, miR-322/-503 cluster was found to be abundantly expressed in the earliest mice cardiac progenitor cells, it induced cardiac and skeletal muscle cell differentiation but inhibited neural lineages, which is likely mediated by targeting the RNA-binding protein CUG-binding protein Elav-like family member 1 (Shen et al., 2016). Taken together, miR-503 is a regulator in cell differentiation.

### miR-503 Expression Level and Its Molecular Targets During Cell Proliferation and Apoptosis

miRs have been demonstrated to regulate cell proliferation and apoptosis. Here, studies of miR-503 in regulating cell proliferation or apoptosis *in vitro* are summarized and listed in Table 1.

**TABLE 1:** Roles of miR-503 in cells and its targets.

miRs	Cell types	Identified targets	References
miR-503-5p	Human aortic SMCs	INSR	Bi et al., (2016)
miR-503-5p	ECSCs	Cyclin D1, Bcl-2, VEGFA and ROCK1	Hirakawa et al., (2016)
miR-503-5p	EPCs	Apelin	Wen et al., (2018)
miR-503-5p	BMDCs	Bcl-2	Min et al., (2013)
miR-503-3p	Lung cancer cells	p21	Sun et al., (2017)
miR-503-5p	Podocytes	E2F3	Zha et al., (2019)
miR-503-5p	HMEC	Apelin-12	Chen et al., (2020)

In human aortic smooth muscle cells (SMCs), treatment with platelet-derived growth factor (PDGF) reduced miR-503 expression level significantly in a dose and time-dependent manner, while overexpression of miR-503 inhibited SMCs proliferation and migration induced by PDGF, miR-503 played this role by downregulating its target insulin receptor (Bi et al., 2016). Carotid artery stenosis (CAS) is a kind of atherosclerotic vascular disease, and the abnormal cell proliferation of vascular SMCs is related with the occurrence of CAS (Faxon et al., 2004). Yan et al., (2020) reported that plasma level of miR-503 in asymptomatic patients with CAS was lower compared with that in healthy individuals, and they also demonstrated that overexpression of miR-503 in vascular SMCs *in vitro* inhibited cell proliferation, and miR-503 inhibitor counteracted this effect. It is concluded that miR-503 can be a potential diagnostic marker for CAS and that miR-503 overexpression may improve CAS by inhibition of cell proliferation of vascular SMCs (Yan et al., 2020). However, Cremer et al., (2019) reported that long noncoding RNA (lncRNA) MALAT1 (metastasis-associated lung adenocarcinoma transcript 1) expression level was reduced in atherosclerotic vessels of symptomatic patients, and that transplantation of MALAT1-deficient bone marrow cells in apolipoprotein E-deficient (Apoe^−/−^) mice promoted atherosclerosis *in vivo*, the MALAT1 targets including miR-503 in Apoe^−/−^ Malat1^−/−^ bone marrow cells were increased. In animal model of atherosclerosis, miR-503 was also increased in aortic roots of Apoe^−/−^ Malat1^−/−^ mice, and inhibition of miR-503 *in vitro* in bone marrow mononuclear cells from Apoe^−/−^ Malat1^−/−^mice reduced cell adhesion to endothelial cells pre-stimulated by tumor necrosis factor-α (TNF-α). However, whether overexpression or inhibition of miR-503 can alleviate atherosclerosis needs further studies *in vivo*.

In human endometriotic cyst stromal cells (ECSCs) (Hirakawa et al., 2016), the expression level of miR-503 was downregulated due to DNA hypermethylation, and miR-503 overexpression in ECSCs resulted in inhibition of cell proliferation, induction of cell apoptosis and reduction of extracellular matrix (ECM) contractility by suppressing its targets cyclinD1, B-cell lymphoma/leukemia-2 (Bcl-2), Ras homology A (RhoA), Rho-associated coiled-coil-forming protein kinase (ROCK1), ROCK2 and vascular endothelial growth factor A (VEGFA) (Hirakawa et al., 2016). In murine bone marrow-derived dentritic cells (BMDCs) (Min et al., 2013), some miRs including miR-503, miR-21, miR-155, miR-146a, miR-142, miR-22 and miR-16-1 were elevated when BMDCs were cocultured with tumor cells, and miR-503 downregulated the expression level of its target Bcl-2 at transcript and protein levels, leading to BMDCs apoptosis, while inhibition of miR-503 prolonged BMDCs lifespan, and thus may improve antitumor response in BMDCs.

Under hypoxia condition, the role of miR-503 is inconsistent in different cell types (Keighron et al., 2018; Wen et al., 2018). For instance, Endothelial progenitor cells (EPCs) are a kind of blood cells which have the characteristics of vascular regeneration (Keighron et al., 2018). In mouse bone marrow derived EPCs, miR-503 was downregulated under hypoxia condition (Wen et al., 2018), and its overexpression inhibited cell proliferation, migration and angiogenesis of EPCs via regulating its target Apelin, which is encoded by *APLN* gene. *APLN* gene encodes preproapelin, then preproapelin is cleaved to several apelin peptides including apelin-36, -17, -12, and -13 (Wysocka et al., 2018). However, in bone marrow-derived mesenchymal stem cells, Nie et al., (2011) reported that miR-503 was upregulated following 6 h of hypoxia stimulation, inhibition of miR-503 aggravated mesenchymal stem cells apoptosis.

In non-small cell lung cancer (NSCLC) cell lines, Sun et al., (2017) demonstrated that overexpression of miR-503-3p suppressed cell proliferation and induced cell apoptosis via downregulating its target p21. p21 is one of cyclin dependent kinase inhibitor proteins (CDKIs) which include Ink4 family and KIP family (Watanabe et al., 1998). However, Chen et al., (2017) reported that long non-coding RNA SNHG20 promoted cell proliferation of NSCLC cell lines partly by downregulating p21 expression. Zhang and Yan (2012) reported that carboxylated multiwalled carbon nanotubes (MWCNTs) treatment inhibited cell proliferation and induced p21 protein overexpression in cells without detectable DNA damage and apoptosis, and the upregulated p21 protein inhibited the function of cyclin D/cdk4,6 complex, then inactivated retinoblastoma (Rb) phosphorylation leading to cell cycle arrest at G1/S phase. In mouse embryonic fibroblasts p21 ^−/−^ cells, MWCNTs treatment didn’t induce cell cycle arrest at G1/S phase (Zhang and Yan, 2012). From Sun Y et al.’s study (Sun et al., 2017), it is inferred that the downregulation of p21 mediated by miR-503-3p in NSCLC cell lines can inhibit cell proliferation and promote cell apoptosis. However, Chen ZY et al.’s study (Chen et al., 2017) indicated that the downregulation of p21 mediated by SNHG20 in NSCLC cell lines promoted cell proliferation. While Zhang Y et al.’s study (Zhang and Yan, 2012) inferred that upregulation of p21 can inhibit cell proliferation. Thus, p21 has a dual role in cell proliferation and apoptosis, and this dual role depends on its cellular localization, and p21 regulation of cell proliferation and apoptosis has been extensively reviewed elsewhere (Kreis et al., 2019; Manu et al., 2019). The inconsistent role of p21 mediated by miR-503-3p or SNHG20 in NSCLC cell lines may be due to the different cell lines used. However, the inconsistency from these studies needs to be investigated further.

In podocytes, high glucose (HG) treatment increased miR-503 expression level in a time and dose-dependent manner and decreased E2F transcription factor 3 (E2F3) expression level (Zha et al., 2019). miR-503 overexpression increased podocytes apoptosis, while its inhibition improved renal function in rats of Diabetes mellitus via targeting E2F3 (Zha et al., 2019). In human microvascular endothelial cells (HMEC-1) (Chen et al., 2020), HG treatment also increased miR-503 expression level markedly, but decreased the expression of Apelin-12, which was proven to be a miR-503 target by luciferase assay. miR-503 inhibited Apelin-12 expression, while it up-regulated the phosphorylation of JNK and p38MAPK. In contrast, inhibition of miR-503 alleviated oxidative stress, inflammation and apoptosis induced by HG in HMEC-1 cells (Chen et al., 2020). The expression level of miR-503 induced by HG in both types of cells is consistent, and the increase of miR-503 contributes to cell apoptosis under HG condition.

### Roles of miR-503 in Carcinogenesis

Accumulated evidence has shown that miR-503 is implicated in carcinogenesis and angiogenesis. Uncontrolled angiogenesis contributes to tumor growth and invasiveness (Peter and Rakesh, 2011). miR-503 expression is dysregulated in tumor or cancer. In this section, we summarize the related findings of miR-503 in cancer and list in Table 2. The role of miR-503 in angiogenesis will be discussed in the next section.

**TABLE 2:** The expression level and targets of miR-503 in cancer.

miRs	Cancer types	Upregulated↑/downregulated↓	Identified targets	References
miR-503-5p	HCC	↓	FGF2, VEGFA	Zhou et al., (2013)
miR-503-5p	HCC	↓	Cyclin D3, E2F3	Xiao et al., (2013)
miR-503-5p	NSCLC	↓	PI3K p85, IKK-β	Yang et al., (2014)
miR-424/-503	Breast cancer	NA	Smad7, Smurf2	Li et al., (2014)
miR-503-5p	Breast cancer	↓	IGF-1R	Yan J. et al., (2017)
miR-503-5p	Breast cancer	↓	CCND1	Long et al., (2015)
miR-503-3p	Breast cancer	↑	Smad2, E-cadherin	Zhao et al., (2017)
miR-503-5p	CC	↓	AKT2	Fu et al., (2019)
miR-503-5p	Prostate cancer	↓	ZNF217	Jiang et al., (2016)
miR-503-5p	GBM	↓	IGF-1R	Zhang et al., (2014)
miR-503-5p	GBM	↑	PDCD4	Guo et al., (2017)
miR-503-5p	CRC	↓	E2F3	Chang et al., (2015)
miR-503-5p	CRC	↑	CaSR	Noguchi et al., (2016)
miR-503-5p	GC	↓	HMGA2	Li et al., (2019)
miR-503-5p	Osteosarcoma	↓	IGF-1R	Wang et al., (2017)
miR-503-5p	ESCC	↑	PRKACA	Wu et al., (2018)
miR-503-5p	Retinoblastoma	↑	PTPN12	Cheng and Liu (2019)

NA, Not applicable.

PI3K/Akt signaling pathway plays an important role in cell growth and cell survival (Cantley, 2002). Phosphionositide 3-kinaes (PI3Ks) consists of catalytic subunits and regulatory subunits, and PI3Kp85 is one of the regulatory subunits (Fruman et al., 1998). In human NSCLC, miR-503 expression level was downregulated in lung cancer tissues compared with adjacent non-cancerous tissues (Yang et al., 2014), while its overexpression impeded tumor growth, metastasis and cell proliferation in a xenografted mice model, and in NSCLC cell lines. Both PI3Kp85 and IKK-β were proven as direct targets of miR-503 by luciferase assay (Yang et al., 2014). IκB kinase β (IKK-β) has been shown to link inflammation to cancer, and deletion of IKK-β can reduce colitis-associated tumor incidence and attenuate tumor growth (Greten et al., 2004). In NSCLC, miR-503 might act as a tumor suppressor by downregulating its targets PI3K p85 and IKK-β (Yang et al., 2014).

In human hepatocellular carcinomas (HCC) tumor, two studies have consistently reported that miR-503 expression level was downregulated in tumor tissues compared with matched adjacent normal tissues (Xiao et al., 2013; Zhou et al., 2013), and miR-503 was also downregulated in HCC cell lines compared with normal liver cell line L02 (Xiao et al., 2013), its down-regulation may be due to epigenetically modulation of the promoter of miR-503 (Zhou et al., 2013). The down-regulation of miR-503 was correlated with the low survival rate of HCC patients (Xiao et al., 2013). Overexpression of miR-503 impeded tumor growth and angiogenesis in a hepatoma xenograft mouse model (Zhou et al., 2013). *In vitro*, miR-503 overexpression repressed human umbilical vein endothelial cells (HUVECs) capillary tube formation, HCC cell proliferation and colony formation, and induced cell cycle arrest at G1 phase in HCC cells through inhibiting its targets, fibroblast growth factor-b (FGF2), VEGFA, cyclin D3 and E2F3 (Xiao et al., 2013; Zhou et al., 2013).

In breast cancer, the reports about miR-503 expression level from different cancer stages are inconsistent. Li et al., (2014) found that miR-424/-503 cluster was significantly higher in breast cancer tissues from patients with metastasis than that without metastasis. Smad7 and Smurf2 are inhibitory factors of transforming growth factor beta (TGF-β), and the targets of miR-424/-503 cluster. Overexpression of this cluster suppressed Smad7 and Smurf2 expression and activated TGF-β signaling, promoting breast cancer cells metastasis in nude mice, while inhibition of miR-424/-503 cluster in breast cancer cells attenuated metastasis in nude mice and improved host survival, suggesting that miR-424/-503 cluster act as “onco-miR” in breast cancer. However, in another two studies, miR-503 in cancer tissues or cells was shown to be reduced remarkably compared to adjacent non-cancerous tissues and non-malignant breast epithelial cells (Long et al., 2015; Yan J. et al., 2017). Moreover, miR-503 was lower in T2-T4 stage of breast cancer than that in T1 stage (Yan J. et al., 2017). Overexpression of miR-503 impeded breast cancer cell proliferation and invasion through inhibiting its target genes Insulin-like growth factor 1 receptor (IGF-1R) (Yan J. et al., 2017) and CCND1 (encoding cyclin D1) (Long et al., 2015). IGF-1R belongs to IGF receptor family, and IGF-1R signaling pathway participates in tumor growth and metastasis (Tognon and Sorensen, 2012). Therefore, the inconsistence of miR-503 expression level in different stages of breast cancer needs more evidences, the effects of its overexpression or inhibition in breast cancer on metastasis deserve further investigation.

In breast cancer, miR-503-3p expression level was found to be higher in cancer tissues and plasma of patients compared with adjacent normal tissues and plasma of healthy subjects, and the high level of miR-503-3p was correlated with breast cancer metastasis (Zhao et al., 2017). Smad2 and E-cadherin were proven as miR-503-3p targets by luciferase assay, and the mRNA levels of both Smad2 and E-cadherin were lower in cancer tissues of patients compared with adjacent normal tissues, and it has been demonstrated *in vitro* that miR-503-3p promotes Epithelial-mesenchymal transition (EMT) and tumor metastasis by downregulation of Smad2 and E-cadherin. Tumor metastasis is a complicated process, it contains EMT and Mesenchymal-epithelial transition (MET). E-cadherin is one of EMT markers, and loss of this protein promotes metastasis (Heerboth et al., 2015). Smads family are intracellular effectors of the TGF-beta superfamily, and Smad2 is one member of Receptor Activated Smads family (Hanna et al., 2021).

In cervical cancer (CC), the expression level of miR-503 in cancer tissues of patients was downregulated compared with adjacent normal tissues, and overexpression of miR-503 in CC cell lines inhibited cell proliferation and colony formation by targeting AKT2, while miR-503 inhibitor reversed these effects (Fu et al., 2019). The mRNA expression level of AKT2 is higher in cancer tissues than that in adjacent normal tissues, and it was proven as miR-503 target by luciferase assay. AKT2 is one isoform of AKT (also called protein kinase B), and activation of AKT promotes cell proliferation and survival (Osaki et al., 2004). Thus, miR-503 may act as a tumor suppressor in CC (Fu et al., 2019). In prostate cancer, miR-503 expression level was also downregulated in cancer tissues of patients compared with adjacent normal tissues (Jiang et al., 2016), its down-regulation was associated with invasive features and poor prognosis in prostate cancer patients. GATA binding protein 3 (GATA3) is a zinc-binding transcription factor, it is found to bind the promoter of miR-503 and activate miR-503 transcription, however, GATA3 expression was also decreased remarkably in prostate cancer tissues. Zinc finger protein 217 (ZNF217) was proven as miR-503 target, it is demonstrated that GATA3/miR-503 axis suppressed the progression of prostate cancer by inhibiting ZNF217 expression.

In human glioblastoma multiforme (GBM), Zhang et al., (2014) found that miR-503 expression level was reduced markedly in GBM tissues and cells compared with human normal brain tissues from patients with cerebral trauma, its overexpression in GBM cells impeded cell proliferation, migration and tumor invasion, and increased cell apoptosis. They further demonstrated that miR-503 acted as tumor suppressor in GBM partially by downregulating its target IGF-1R and interfering with PI3K/AKT pathway. However, Guo et al., (2017) found that the expression level of miR-503 was elevated in glioblastoma tissues or cancer cells compared to normal human brain tissue and normal human astrocytes separately. And they showed that overexpression of miR-503 in glioblastoma cells increased cell proliferation, and reduced cell apoptosis by targeting PDCD4 (programmed cell death 4). By contrast, inhibition of miR-503 impeded cell proliferation (Guo et al., 2017). PDCD4 is known as one tumor suppressor gene which inhibits cell proliferation, tumor angiogenesis and induces apoptosis (Lankat-Buttgereit and Goke, 2009). Zhang et al., (2014) compared miR-503 expression level in cancer cells with that in normal brain tissues and found that miR-503 was downregulated, Guo et al., (2017) did not describe clearly from where the normal human brain tissue was obtained. Thus, the inconsistent expression level of miR-503 between these two studies may be due to the source of control samples. Also, the function of miR-503 in glioblastoma between both studies is inconsistent, this needs to be investigated further.

In colorectal cancer (CRC), the role of miR-503 is also inconsistent. Chang et al., (2015) reported that miR-503 expression level was reduced remarkably in cancer tissues or cells compared with adjacent normal tissues and normal colonic cell line separately, its overexpression in CRC cells suppressed cell proliferation and increased cell apoptosis rate by targeting E2F3. Whereas in Noguchi et al.’s study (Noguchi et al., 2016), in normal mucosa, colorectal adenoma and cancer tissues, the expression level of miR-503 was increased in sequence from adenoma to carcinoma, higher miR-503 expression in patients was related with large tumor size, tumor invasion and metastasis, and inhibition of miR-503 in CRC cells suppressed cell proliferation, invasion and migration, which is suggestive that miR503 acted as an “onco-miR” in CRC by downregulation of calcium-sensing receptor (CaSR) expression. CaSR is a G protein-coupled receptor and functions as a tumor suppressor in colon cancer (Singh et al., 2013). Therefore, the expression level and function of miR-503 in CRC is inconsistent. In Noguchi et al.’s study, the control samples were from healthy volunteers, while in Chang et al.’s study, the control samples were from adjacent normal tissues of cancer patients, this may explain the inconsistence of miR-503 expression. Nonetheless, the function of miR-503 in CRC deserves further investigation.

In other gastrointestinal cancer, such as gastric cancer (GC) (Li et al., 2019) and esophageal squamous cell carcinoma (ESCC) (Wu et al., 2018), the miR-503 expression level is converse. miR-503 expression decreased remarkably in tissues of GC, but highly expressed in tissues of ESCC, as compared with individual adjacent non-cancerous tissues (Wu et al., 2018; Li et al., 2019), while it was lower in tissues of ESCC with metastasis than that without metastasis (Wu et al., 2018). In GC, overexpression of miR-503 impeded cell proliferation, invasion and colony formation *in vitro* and tumor growth *in vivo* by suppressing target gene *HMGA2* (encoding High-mobility group AT-hook 2) and WNT/β-catenin signaling pathway (Li et al., 2019). Ectopic expression of miR-503 in ESCC cells also impeded cell proliferation, invasion and metastasis by inducing cellular autophagy *in vitro* and *in vivo*. Protein kinase cAMP-activated catalytic subunit alpha (PRKACA) was proven as miR-503 target gene by luciferase assay, and it is suggested that miR-503 induced autophagy in ESCC cells via the protein kinase A (PKA)/mammalian target of rapamycin (mTOR) pathway (Wu et al., 2018).

In osteosarcoma, miR-503 expression level was reduced in tumor tissues compared with matched adjacent normal tissues, and it was lower in specimens from patients of T3-T4 stage than that of T1-T2 stage (Wang et al., 2017). *In vitro,* miR-503 was also lower in osteosarcoma cells than that in normal osteoblasts cells, and overexpression of miR-503 suppressed the proliferation and invasion of osteosarcoma U2OS cells, thus, it acted as a tumor suppressor in osteosarcoma via inhibiting its target, IGF-1R. However, in retinoblastoma (RB) (Cheng and Liu, 2019), miR-503 was greatly expressed in RB tissues, and acted as an “onco-miR” by targeting PTPN12.

Taken together, miR-503 expression level varies in different types of cancer. However, except for the inconsistent results of colorectal cancer and glioblastoma multiforme, miR-503 has been found to be downregulated in most of cancer types, but upregulated in few cancer types, such as retinoblastoma (Zhao et al., 2009; Cheng and Liu, 2019), ESCC (Wu et al., 2018) and adrenocortical carcinoma (Ozata et al., 2011). miR-503 can act as “onco-miR” or tumor suppressor in different types of cancer. The expression level and targets of miR-503 in cancer are listed in Table 2.

### Roles of miR-503 in Angiogenesis

Numerous miRNAs, such as miR-92a (Bonauer et al., 2009), miR-29b (Li et al., 2017), and miR-21 (Liu et al., 2016), are demonstrated to regulate angiogenesis, and have the characteristics of anti- or pro-angiogenesis. The miR-16 family in angiogenesis and diabetes has been reviewed elsewhere (Caporali and Emanueli, 2011). In this section, we focus on studies of miR-503 in angiogenesis.

The role of miR-503 in angiogenesis has been demonstrated *in vitro* and *in vivo* (Caporali et al., 2011; Jongmin et al., 2012; Zhou et al., 2013; Caporali et al., 2015; Wen et al., 2018). In EPCs (Wen et al., 2018), miR-503 suppressed angiogenesis by targeting Apelin. In Diabetes mellitus, miR-503 expression level was significantly higher in ischemic muscles and plasma from patients with limb ischemia than normal control (Caporali et al., 2011), and it is also higher in the plasma of diabetic patients with ischemic stroke than that in non-diabetic patients with stroke or in control individuals (Sheikhbahaei et al., 2019). Overexpression of miR-503 suppressed endothelial cells proliferation, migration and network formation by suppression of its targets, cdc25A (cell division cycle 25 A) and CCNE1 (cyclin E1) under normal culture condition (Caporali et al., 2011). In diabetic mice with limb ischemia, antagonizing miR-503 improved angiogenesis and blood flow recovery by upregulating cdc25A and CCNE1 (Caporali et al., 2011). Furthermore, it was found that p75 neurotrophin receptor (p75 ^NTR^) activated NF-κB to bind the promoter of miR-503 and activated its expression in endothelial cells exposed to high glucose (Caporali et al., 2015). p75^NTR^ is a multifunctional membrane receptor of nerve growth factor (Caporali and Emanueli, 2009).The microparticles carrying miR-503 were shed from diabetic endothelial cells and transferred into recipient pericytes, where miR-503 inhibited pericyte migration and proliferation by targeting *EFNB2* (encoding Ephrin-B2) and *VEGFA*, and subsequently increased vascular permeability and blocked angiogenesis in post-ischemic limb muscles of diabetic mice (Caporali et al., 2015).

In hepatocellular carcinomas (Zhou et al., 2013), miR-503 inhibited tumor angiogenesis by down-regulation of FGF2 and VEGFA. The VEGF family and FGF superfamily are regulators in angiogenesis (Peter and Rakesh, 2011), VEGF (also known as VEGFA) is a predominant regulator in the process of angiogenesis, which stimulates angiogenesis via VEGF receptor-2, thus, blockers of VEGF is widely used as an anti-angiogenic agent (Peter and Rakesh, 2011). In glioma cells, overexpression of miR-503 reduced both LRIG2 (Leucine-rich repeats and immunoglobulin-like domains protein 2) and VEGFA expression levels (Sun et al., 2019), and suppressed angiogenesis in cocultured human cerebral microvascular endothelial cell line D3 (HCMEC/D3), while miR-503 inhibitor promoted angiogenesis in cocultured HCMEC/D3. LRIG2 was proven as miR-503 target by luciferase assay, knockdown of LRIG2 reduced VEGFA expression level along with the condition of miR-503 inhibitor, therefore, miR-503 regulated angiogenesis in glioma by reducing LRIG2 expression followed by downregulation of VEGFA expression (Sun S. et al., 2019). LRIG2 belongs to the leucine-rich repeats and immunoglobulin-like domains family and regulates epidermal growth factor receptor (EGFR) signaling pathway (Simion et al., 2014), and downregulation of LRIG2 suppressed angiogenesis in glioma (Yang et al., 2017). In pulmonary arterial hypertension (Jongmin et al., 2012), both hsa-miR-424 and hsa-miR-503 were decreased in pulmonary artery endothelial cells derived from patients with pulmonary arterial hypertension, and overexpression of both miRs in animal models of pulmonary hypertension can alleviate pulmonary hypertension by downregulation of FGF2 and FGF receptor (FGFR1) (Jongmin et al., 2012).

In chronic obstructive pulmonary disease (COPD) (Jun et al., 2017), miR-503 was decreased in lung fibroblasts from COPD patients, VEGF released by lung fibroblasts from patients with COPD were higher compared with that from patients without COPD, it is inferred that miR-503 may regulate fibroblast-mediated vascular homeostasis in COPD via VEGF signaling, given that VEGF plays an important role in regulating vascular homeostasis in various pathological conditions (Tsai et al., 2009; Liu et al., 2019a). In another clinical trial (Fei et al., 2018), patients with coronary artery disease (CAD) were divided into good coronary collateral circulation (CCC) formation group and poor CCC formation group, and the level of miR-503 in the plasma from good CCC or poor CCC group was lower compared with that from control subjects, and miR-503 was negatively correlated with CCC formation and VEGFA (Fei et al., 2018). This indicates that miR-503 might suppress CCC formation, and the downregulation of miR-503 in CAD patients may be a compensatory effect of protection.

Overall, the role of miR-503 in angiogenesis is consistent in different studies, and it shows the characteristic of anti-angiogenesis.

### Roles of miR-503 in Tissue Fibrosis

Fibrosis can a significant pathological state in many organs, such as heart (Gao et al., 2012; Liu et al., 2017; Ge Z. D. et al., 2019), lung (Yang et al., 2020), kidney and liver, and may result in the distortions of organ structure and organ dysfunction (Li et al., 2016; Warisara et al., 2020). Pro-fibrotic mediator TGF-β (Guo et al., 2010) and its signaling pathways play an important role in the process of epithelial-mesenchymal transition and tissue fibrosis (Li et al., 2016; Warisara et al., 2020). Multiple miRs are involved in fibrosis, for instance, miR-21 stimulated the development of lung fibrosis (Liu et al., 2010), while miR-101 inhibited cardiac fibrosis after myocardial infarction (MI) (Pan et al., 2012). Several studies reported that miR-503 was implicated in fibrosis (Zhou et al., 2016; Yan W. et al., 2017), in this section, we list the alterations of miR-503 in fibrosis and discuss how miR-503 regulates fibrosis in lung or heart.

Cardiac fibrosis can lead to contractile dysfunction (Xia et al., 2007; Gao et al., 2012; Li et al., 2015) and cardiac arrhythmia (Warisara et al., 2020). In mouse heart tissues of cardiac fibrosis induced by transverse aortic constriction (TAC) and mouse neonatal cardiac fibroblasts (CFs) cultured with Angiotensin II (Zhou et al., 2016), miR-503 expression level was upregulated, its overexpression in neonatal CFs increased cell proliferation and collagen production mediated by Angiotensin II, while pretreatment with Apelin-13 and co-transfection of miR-503 in neonatal CFs resulted in inhibition of TGF-β and connective tissue growth factor (CTGF) expression and collagen production, Apelin-13 treatment alone decreased cell proliferation and collagen production, and reduced TGF-β and CTGF expression levels in neonatal CFs mediated by Angiotensin II (Zhou et al., 2016). Apelin-13 was identified as target of miR-503 by luciferase assay, and antagomiR-503 treatment in TAC mice improved cardiac function and suppressed both TGF-β and CTGF expression, therefore, miR-503 has the property of promoting cardiac fibrosis in TAC mouse model. In contrary, in mouse model of pulmonary fibrosis induced by silica (Yan W. et al., 2017), miR-503 was reduced in fibrotic lung tissue and cells exposed to silica compared with control, but long non-coding RNA MALAT1 was upregulated, and MALAT1 regulated miR-503 expression by competitively binding to miR-503. Snail is a transcription factor and recognized as inducer of EMT (Kalluri and Weinberg, 2009). PI3K p85 was also proven as miR-503 target, and the activation of PI3Kp85/Akt/mTOR/Snail pathway facilitated the development of EMT and pulmonary fibrosis (Yan W. et al., 2017), overexpression of miR-503 has been shown to inhibit silica-induced pulmonary fibrosis by inactivating that pathway (Yan W. et al., 2017).

From those studies, it is inferred that miR-503 attenuates pulmonary fibrosis, while it promotes cardiac fibrosis, thus the findings regarding the function of miR-503 in fibrosis is contradictory, which may be due to differences in the process of pulmonary fibrosis and cardiac fibrosis. EMT plays an critical role in the pathogenesis of pulmonary fibrosis (Heise et al., 2011), some epithelial cells in the lung can be transformed to fibroblasts by EMT (Kim et al., 2006). It is notable that there are no epithelial cells in the heart tissue, however, endothelial-to mesenchymal transition (EndMT) can be induced in the heart after MI, and EndMT becomes the source of myofibroblasts and promotes the production of fibroblastic content (Aisagbonhi et al., 2011).The molecular mechanism whereby miRs regulate tissue fibrosis formation is complicated and needs further study.

### Roles of miR-503 in Oxidative Stress

Oxidative stress occurs when excessive generation of reactive oxygen species (ROS) exceeds antioxidant defense systems (González-Montero et al., 2018; Liu et al., 2020a; Liu et al., 2020b). ROS include superoxide anion (O_2_^−^), hydroxyl radicals (^.^OH), peroxynitrite anion (ONOO^−^) and hydrogen peroxide (H_2_O_2_), they can cause oxidative damage to the lipids, proteins, and nucleic acids of cells (González-Montero et al., 2018; Li et al., 2018; Lei et al., 2019). To remove ROS, cells use intrinsic antioxidant defense systems, including antioxidative enzymes and antioxidants, such as superoxide dismutase (SOD), catalase (CAT), glutathione peroxidase (GPx), and glutathione, to protect against the injurious effects of excessive ROS (Finkel, 2003; Cai et al., 2017; González-Montero et al., 2018). Hyperglycemia (Rolo and Palmeira, 2006; Yang et al., 2015), hypoxia (Deng et al., 2017; Tang et al., 2018), ischemia or ischemia/reperfusion (I/R) (Yu et al., 2015; González-Montero et al., 2018), all induce oxidative stress.

Multiple miRs have been shown to be involved in the development of oxidative stress, this topic has been reviewed elsewhere (Magenta et al., 2013; Nikolai et al., 2018; Carbonell and Gomes, 2020), and the miRs that have been reported to regulate antioxidant system are listed in the latest review (Carbonell and Gomes, 2020). Some miRs can aggravate oxidative stress, whereas others have the opposite roles. For instance, the application of miR-223-3p inhibitor can alleviate hypoxia-induced oxidative stress and apoptosis (Tang et al., 2018), while miR-377 inhibitor can alleviate renal I/R-induced oxidative stress and inflammation (Liu et al., 2019b). Both miR-27a (Zhao Y. et al., 2018) and miR-140-5p (Zhao L. et al., 2018) can exacerbate oxidative damage by down-regulation of the target Nrf2 (nuclear factor erythroid 2-related factor 2). Nrf2 is an important transcription factor which binds to the antioxidant response element (ARE) (Kensler et al., 2007), and regulates key genes involved in oxidative stress, such as SOD, CAT, heme oxygenase 1 (HO-1) and glutamate-cysteine ligase modifier subunit (GCLM), while Nrf2 expression is also regulated by its inhibitory protein called Kelch-like ECH-associated protein 1 (Keap1) and plays a critical role in promoting endogenous antioxidant capacity to combat ischemia-reperfusion injury induced oxidative stress and organ or tissue injuries (Gao et al., 2016; Han et al., 2016; Luo et al., 2019). By contrast, miR-7 can target Keap1 and activate Nrf2 pathway, therefore, it can relieve oxidative stress and exert cytoprotective effects by regulating the Nrf2 pathway (Kabaria et al., 2015). Several studies have reported that miR-503 is involved in oxidative stress (Miao et al., 2017; Rubattu et al., 2017; Chen et al., 2020; Zhang et al., 2020). In this section, we summarize and analyze the mechanisms of miR-503 in oxidative stress.

Myocardial I/R injury can lead to oxidative stress in both the experimental settings of myocardial I/R (Xia et al., 2003a; Xia et al., 2003b; Zhengyuan et al., 2005; Liu et al., 2011) and in patients undergoing open heart surgeries under cardiopulmonary bypass (Ansley et al., 2003; Xia et al., 2006; Huang et al., 2011). Varga et al., (2014) reported that miR-503 was downregulated in the isolated rat hearts subjected to 30 min of left anterior descending coronary artery ligation followed by 120 min of reperfusion, and ischemic postconditioning attenuated the downregulation of miR-503. However, the exact role of miR-503 in oxidative stress induced by I/R has not been explored yet. In diabetic cardiomyopathy (DCM) (Miao et al., 2017), miR-503 was increased significantly in the rat heart tissues but decreased by Phase II enzyme inducer (CPDT) treatment. Nrf2 was proven as miR-503 target by luciferase assay, it was decreased in DCM but increased by CPDT treatment (Miao et al., 2017). It is suggested by Miao et al., (2017) that CPDT can alleviate oxidative stress in DCM through miR-503/Nrf2/ARE signaling pathway, while the authors did not simultaneously detect the oxidative stress markers in the presence of either the miR-503 mimic or an inhibitor of miR-503 in diabetic rats, nor in cultured cardiomyocytes exposed to high glucose mimicking the situation of diabetes. In human microvascular endothelial cells (Chen et al., 2020), miR-503 inhibitor alleviated HG-induced oxidative stress by the inactivation of JNK and p38 MAPK phosphorylation, suggesting that miR-503 has the property of pro-oxidative stress.

In spontaneous hypertension and stroke rat model (Rubattu et al., 2017), the miR-503 expression level was increased significantly in brains of Spontaneously Hypertensive Stroke Prone rats (SHRSP) upon high-salt diet, but reduced in brains of Spontaneously Hypertensive Stroke Resistant Rats (SHRSR). The peroxidation end products carbonylated total proteins were also increased markedly in brains of high-salt-fed SHRSP, but not in high-salt-fed SHRSR. In contrast, the mRNA and protein expression levels of uncoupling protein 2 (UCP2) were decreased significantly in brains of SHRSP upon high-salt diet, but not in brains of SHRSR. And overexpression of miR-503 in endothelial cells *in vitro* decreased UCP2 expression and increased cell mortality (Rubattu et al., 2017). It is indicated that the downregulation of UCP2 expression regulated by miR-503 increases oxidative stress and stroke occurrence in high-salt-fed SHRSR. UCP2 is a mitochondrial membrane protein and wildly expressed among organs (Mattiasson and Sullivan, 2006), and overexpression of UCP2 protected against endothelial dysfunction of vessels through reducing ROS production followed by increasing nitric oxide (NO) bioavailability (Tian et al., 2012). Another study reported that miR-503 expression level in plasma of patients with moderate and severe ischemic stroke was highest compared to patients with minor stroke and control individuals (Zhang et al., 2020). It is also demonstrated that overexpression of miR-503 increased cell apoptosis and ROS production, but reduced NO generation *in vivo* and *in intro*, while inhibition of miR503 alleviated apoptosis, oxidative stress, and increased NO generation by activation of PI3K/AKT/eNOS pathway, and it is indicated that plasma miR-53 may be an attractively diagnostic marker or therapeutic target for ischemic stroke (Zhang et al., 2020).

To summarize, the mechanisms of miR-503 in oxidative stress is not well understood especially in I/R injury or diabetes, it is worthy of further investigation.

### The Mechanisms of Abnormal miR-503 Expression in Cardiovascular Disease or Cancer

**FIGURE 1:**
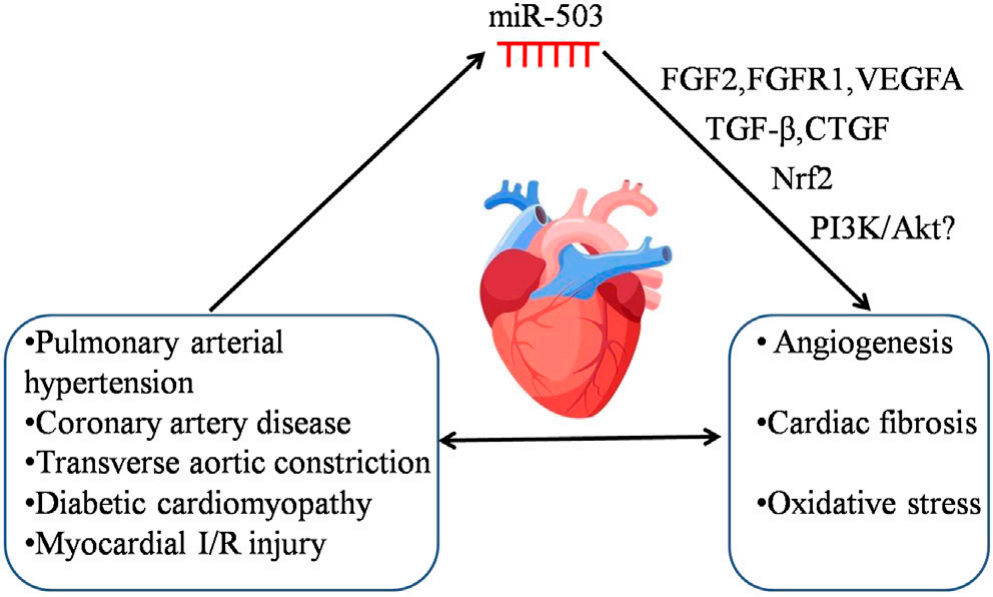
The potential mechanism of miR-503 in regulating cardiovascular disease. Cardiovascular disease induces the abnormal expression of miR-503. The dysregulation of miR-503 may contribute to various pathologies, e.g., angiogenesis, cardiac fibrosis and oxidative stress, and it would subsequently aggravate the severity of cardiovascular disease. The following targets, FGF2, FGFR1, VEGFA, TGF-β, CTGF, Nrf2, and PI3K/Akt may be involved in the pathological processes of cardiovascular disease regulated by miR-503.

Studies have shown that miR-503 is expressed abnormally in human diseases, such as diabetes (Caporali et al., 2011; Xu et al., 2017), pulmonary arterial hypertension, coronary artery disease (Fei et al., 2018), ischemic stroke (Zhang et al., 2020) and cancer, and have explored the roles of miR-503 in diseases by overexpressing or inhibiting miR-503 *in vivo* and *in vitro*. The potential mechanism of miR-503 in regulating cardiovascular disease is shown in Figure 1. However, the mechanisms of its abnormal expression in diseases are little known.

DNA methylation in the promoters of miRs or histone modifications have been evaluated for the dysregulation of miRs (Manel, 2011; Druz et al., 2012; Zhang et al., 2016). The mechanisms governing miR-503 down-regulation or its activation have been explored in few studies. On the one hand, several studies have reported the reasons of miR-503 downregulation. Zhou et al., (2013) and Hirakawa et al., (2016) have shown that the downregulation of miR-503 in HCC tissues or human endometriotic cyst stromal cells may be due to DNA hypermethylation near the promoter of miR-503. Yan W. et al., (2017) reported that lncRNA MALAT1 bound to miR-503 directly as a sponge and downregulated miR-503 expression in silica-induced pulmonary fibrosis. In patients with acute MI and in rat model of myocardial I/R injury, MALAT1 was increased in peripheral blood cells (Vausort et al., 2014) or myocardial tissues (Sun T. et al., 2019). Our unpublished data and Varga et al.’s data (Varga et al., 2014) showed that miR-503 expression level was downregulated in myocardial tissues following I/R injury. However, it is yet to be determined whether or not the downregulation of miR-503 in myocardial I/R injury may be due to the upregulation of MALAT1 or DNA methylation. On the other hand, the mechanism governing the activation of miR-503 expression has also been explored. Kim et al., (Jongmin et al., 2012) demonstrated that overexpression of *APLN* (apelin) induced miR-424/503 expression by regulation of miR-424/503 promoter in pulmonary artery endothelial cells. Jiang et al., (2016) showed that miR-503 expression was activated by GATA3 binding to its promoter. Caporali et al., (2015) showed that NF-κB was activated by p75 neurotrophin receptor (p75 ^NTR^) and bond to the promoter of miR-503, leading to miR-503 transcription in endothelial cells. Taken together, the mechanisms of miR-503 dysregulation are diverse and complicated in different pathological conditions. Further investigation of abnormal miR-503 expression is needed for discovering good therapeutic targets.

## Conclusion and Future directions

miR-503 is extensively studied in cancer, and most studies reported that miR-503 acted as a tumor suppressor, while a few studies showed miR-503 may function as an “onco-miR”. Whether overexpression or inhibition of miR-503 *in vivo* can suppress tumor growth depends on cancer types, and preclinical studies are still needed to address the impact of miR-503 on the development of cancer. In the aspects of tissue fibrosis or oxidative stress, there are few studies of miR-503 and the mechanisms are little known. Cardiac fibrosis can occur following by MI, but it is unclear whether the role of miR-503 in TAC or MI induced cardiac fibrosis is similar. Oxidative stress is one of risk factors in myocardial I/R injury, the role of miR-503 in regulating oxidative stress in myocardial I/R injury is still unknown. To explore miR-503 as therapeutic target or diagnostic marker, further investigation can be focused on the role of miR-503 in tissue fibrosis or oxidative stress, and the dysregulation of miR-503 in cardiovascular disease, especially myocardial I/R injury deserves further investigation.

## Glossary

ARE: antioxidant response element
Bcl-2: B-cell lymphoma/leukemia-2
CTGF: connective tissue growth factor
CAD: coronary artery disease
CAS: Carotid artery stenosis
CC: cervical cancer
CCC: coronary collateral circulation
BMDCs: bone marrow-derived dendritic cells
cdc25A: cell division cycle 25 A
COPD: chronic obstructive pulmonary disease
CRC: colorectal cancer
cdk2: cyclin-dependent-kinase 2
DCM: diabetic cardiomyopathy
ESCC: esophageal squamous cell carcinoma
EMT: epithelial-mesenchymal transition
EPCs: epithelial-mesenchymal transition
ECSCs: endothelial progenitor cells
E2F3: E2F transcription factor 3
FGF2: fibroblast growth factor-b
FGFR1: FGF receptor
GBM: glioblastoma multiforme
GC: gastric cancer
HG: high glucose
HMEC: human microvascular endothelial cells
HCC: hepatocellular carcinomas
IGF-1R: Insulin-like growth factor 1 receptor
I/R: Ischemia/Reperfusion
LRIG2: Leucine-rich repeats and immunoglobulin-like domains protein 2
miRs: microRNAs
MI: myocardial infarction
nt: nucleotides
NSCLC: non-small cell lung cancer
ROS: reactive oxygen species
SMCs: smooth muscle cells
TGF-β: transforming growth factor beta
TAC: transverse aortic constriction
UCP2: uncoupling protein 2
VEGFA: vascular endothelial growth factor A
